# Pediatric outdoor recreational injuries: another hidden concern during the COVID-19 pandemic

**DOI:** 10.1186/s40621-023-00445-6

**Published:** 2023-06-29

**Authors:** Melissa P. Blumberg, Michael A. Gittelman, Wendy J. Pomerantz

**Affiliations:** 1 Division of Emergency Medicine, Department of Pediatrics, Comprehensive Children’s Injury Center, Cincinnati Children’s Hospital Medical Center, University of Cincinnati College of Medicine, 3333 Burnet Avenue, ML #2008, Cincinnati, OH 45229 USA; 2 Division of Emergency Medicine, Pediatric Emergency Medicine, Nemours Children’s Hospital, 1600 Rockland Rd, Wilmington, DE 19803 USA

**Keywords:** Recreation, Injury, COVID-19, Epidemiology

## Abstract

**Background:**

Recreational equipment sales rose significantly during the COVID-19 pandemic. This study investigated changes in the incidence of pediatric emergency department (PED) visits related to outdoor recreational activities during the COVID-19 pandemic.

**Methods:**

A retrospective cohort study was conducted at a large children’s hospital with a level 1 trauma center. Data were obtained from PED electronic medical records of children 5–14 years with a visit from March 23-September 1 in years 2015–2020. Patients with an ICD-10 code for injury associated with recreation and use of common outdoor recreational equipment were included. Initial pandemic year, 2020, was compared with pre-pandemic years (2015–2019). Data collected included patient demographics, injury characteristics, deprivation index, and disposition. Descriptive statistics were used to characterize the population and Chi-squared analysis was used determine relationships between groups.

**Results:**

There were 29,044 total injury visits during the study months with 4715 visits (16.2%) due to recreational mechanisms. A higher proportion of visits due to recreational injury visits occurred during the COVID pandemic (8.2%) compared to before (4.9%). Comparing patients included within the two times, were no differences in sex, ethnicity, or ED disposition. During the COVID pandemic, there was a higher percentage of White patients (80% vs 76%) and patients with commercial insurance (64% vs 55%). There was a significantly lower deprivation index for patients injured during the COVID pandemic. There were more injuries due to bicycles, ATV/motorbike, and non-motorized wheeled vehicles during the COVID pandemic.

**Conclusions:**

During the COVID-19 pandemic, there was an increase in bicycle, ATV/motorbike, and non-motorized wheeled vehicle injuries. White patients with commercial insurance were more likely to be injured compared to years prior. A targeted approach to injury prevention initiatives should be considered.

**Supplementary Information:**

The online version contains supplementary material available at 10.1186/s40621-023-00445-6.

## Introduction

It is well documented that natural disasters, infectious disease outbreaks, and economic crises have affected pediatric injury rates (Rajmil et al. 2015). With the stay-at-home orders during the COVID-19 pandemic, a rising percentage of families turned to socially distanced outdoor activities. Families tended to hike, bike, and play outside to prevent the spread of COVID-19 (http://outdoorindustry.org/article/increase-outdoor-activities-due-covid-19/). Correlating with the demand, sales of outdoor equipment during the COVID-19 pandemic increased by nearly 51% compared to the previous year, for a total sales of close to $193 million dollars (www.npd.com/wps/portal/npd/us/news/press-releases/2020/the-npd-group-sales-of-outdoor-and-sports-toys-surge-as-families-take-refuge-in-their-backyards/). As organized sports, schools, and community gathering places closed, manufacturers sold more playground equipment (+ 81%), skates, skateboards, and scooters (+ 107%), bicycles and other ride-ons (+ 78%)^4^, and pools (+ 24%) (Sciullo 2020; Thompson 2020; Ogletree 2021; Colabine and Justice 2020; Goldbaum 2020).

Emergency departments (EDs) across the country noticed a reduction in overall volumes of patients being seen at the beginning of the COVID-19 pandemic. In accordance with this, though the absolute number of visits related to injury decreased (Johnson et al. 2021a; b; Sanford et al. 2021; Ruzzini et al. 2021; Pines et al. 2021; Wells et al. 2022), the proportion of visits related to injuries increased (Haddadin et al. 2021; O'connor 2020). The mechanisms and types of injuries varied in presentation, with fewer patients presenting for fractures (Ruzzini et al. 2021, Bram et al. 2020), mild traumatic brain injuries (TBIs) (Goldbaum 2020; Kontos et al. 2021), motor vehicle crashes (MVC) and pedestrian injuries (Sanford et al. 2021; Pines et al. 2021; Shi et al. 2021), falls (Sanford et al. 2021; Shi et al. 2021), and sport-related injuries, (Johnson et al. 2021b) and more patients presenting for injuries related to dog bites (Dixon and Mistry 2020; Sethuraman et al. 2021), burns (Sanford et al. 2021), bicycles (Salottolo et al. 2021; Oudtshoorn et al. 2021; Shack et al. 2022), and firearms (Wells et al. 2022; Sethuraman et al. 2021).

Current literature has yet to identify a clear relationship between the pandemic and recreational injuries. The objective of this study was to determine the change in incidence of childhood recreational injuries presenting to a pediatric emergency department (PED) before and during the COVID-19 pandemic. Defining the most common mechanisms of recreational injuries, types of injuries, and the patients at greater risk of injuries during a pandemic is important for future prevention efforts.

## Results

**Fig. 1:**
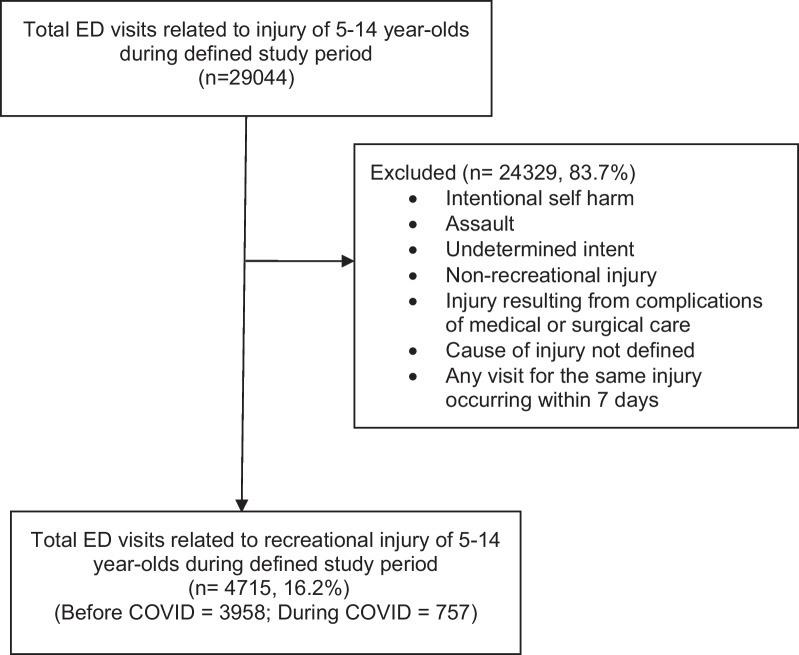
Consort diagram

During the designated study periods, there were a total of 90,010 ED visits, of which 29,044 (32.3%) were due to injury. Of total injury visits, 4715 (16.2%) were the result of an included recreational injury (Fig. 1) with 3958 occurring before the pandemic (average = 792 per year) and 757 occurring during the COVID pandemic. Of the entire study population, more patients were male 2771 (58.8%), White 3614 (76.6%), and with commercial insurance 2668 (56.6%). Comparing data before and during the COVID pandemic, there were no differences in sex, ethnicity, ED disposition or admitting unit (Table 1). Patients presenting to the ED during COVID were older (9.26 years vs 8.79 years, *p* = < 0.001). During the COVID pandemic, there was a higher percentage of White patients (80.1% vs 76.0%, *p* = 0.001) and fewer Black patients (11.8% vs 17.0%, *p* = 0.001) injured in recreational activities. There were also more patients with commercial insurance (64.2% vs 55.1%, *p* < 0.001) injured in a recreational activity during the COVID pandemic compared to the years prior.

**Table 1:** Demographic data (count, %)

	Before pandemic	During COVID pandemic	Total
*Sex*			
Male	2348 (59.3)	423 (55.9)	2771 (58.8)
Female	1610 (40.6)	334 (44.1)	1944 (41.2)
Race*			
White	3008 (76.0)	606 (80.0)	3614 (76.6)
Black	673 (17.0)	89 (11.8)	762 (16.2)
Hispanic	85 (2.1)	26 (3.4)	111 (2.4)
Other/Unknown	192 (4.9)	36 (4.8)	228 (4.8)
Insurance type*			
Commercial	2182 (55.1)	486 (64.2)	2668 (56.6)
Public	1589 (40.1)	244 (32.2)	1833 (38.9)
Self-pay	154 (3.9)	22 (2.9)	176 (3.7)
Other/Unknown	33 (0.8)	5 (0.7)	38 (0.8)
*Hospital disposition*			
Admit	513 (13)	114 (15.1)	627 (13.3)
Discharge home	3430 (86.7)	643 (84.9)	4073 (86.4)
Other	15 (0.04)	0 (0)	15 (0.03)
*Admitting unit*			
Ward	413 (80.5)	98 (85.9)	511 (81.5)
PICU	25 (4.8)	5 (4.4)	30 (4.8)
Psych	5 (0.9)	0 (0)	5 (0.8)
OR	70 (13.6)	11 (9.6)	81 (12.9)

*Statistically significant difference during COVID pandemic compared to the averages of the years prior, *p* < 0.01

**Fig. 2:**
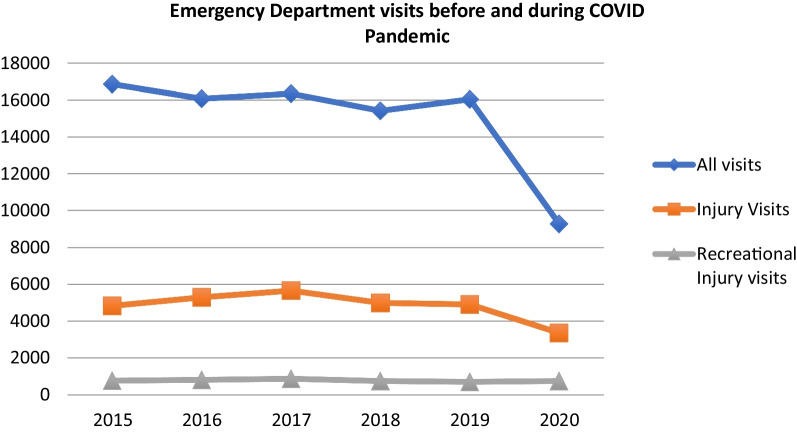
ED visits of 5–14 year-olds from March 23 to Sept 1 in the years before and during COVID

**Fig. 3:**
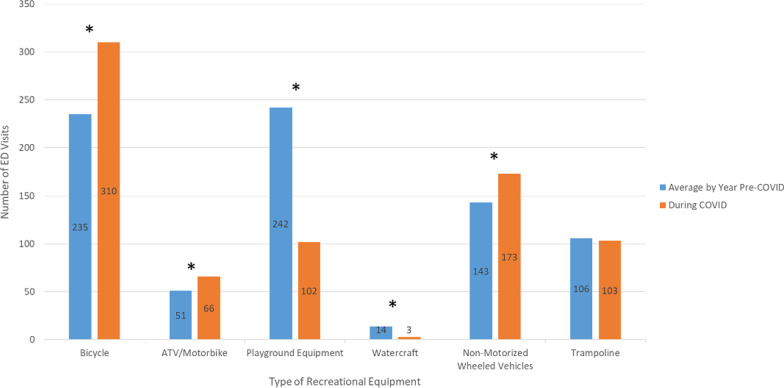
ED visits for recreational injuries by type of recreational equipment before and during the COVID pandemic. *Statistically significant difference during COVID pandemic compared to the averages of the years prior, *p* < 0.001,

During the pandemic, there were fewer overall ED visits and visits related to injury. Despite this, there were a similar number of total visits recreational injury during the pandemic compared to before the pandemic (Fig. 2). As such, there was a higher proportion of visits related to recreational injury during the COVID pandemic (8.2%) compared to before (4.9%), *p* < 0.001. The types of recreational equipment used were significantly different pre- and during the COVID pandemic (*p* < 0.001) (Fig. 3). Specifically, there were significantly more injuries related to bicycles (41% vs 29.7%), ATV/motorbike (8.7% vs 6.4%), and non-motorized wheeled vehicles (22.9% vs 18.1%), *p* < 0.001; there were significant fewer injuries related to playground equipment (13.5% vs 30.5%) and watercraft (0.4% vs 1.8%), *p* < 0.001. There were no significant differences in trampoline injuries. Injury location also differed with significantly more injured at home during the COVID pandemic compared to before (*p* < 0.001). Patients injured during the COVID pandemic were found to be more socioeconomically advantaged than prior based on deprivation index (0.3023 vs 0.3303, *p* < 0.001).

Bicycle injuries represented the greatest number of recreational injuries seen in the population (*n* = 1487, 31.5%). Demographics of bicycle injuries changed during the pandemic, with more White (73.2% vs 69.1%, *p* < 0.001), more female (38.4% vs 32.2%, *p* = 0.04), and more commercially insured patients (33.3% vs 23.5%, *p* < 0.001) presenting with injuries compared to the prior years. Types of bicycle injuries changed during COVID pandemic as well, with more patients who presented for injuries related to fractures (42.3% vs 31.3%) and lacerations (24.5% vs 15.8%), and fewer patients who presented for injuries related to abrasions and contusions (20.6% vs 25.4%), concussions and head injuries (2.3% vs 5.9%), and sprains and strains (1.0% vs 1.6%), *p* < 0.001. During COVID, more patients presented for bicycle injuries of the upper extremities (42.3% vs 33.0%) and fewer patients presented for injuries related to head, neck, and face (29.0% vs 34.4%) and abdomen, thorax, and pelvis (8.7% vs 12.3%), *p* = 0.01.

## Discussion

This data adds to the growing information on injury in pediatrics during the COVID-19 pandemic. Overall these results highlight the increase in the proportion of ED visits related to pediatric recreational injuries during the COVID pandemic. These injuries occurred more often in White and economically advantaged youth.

The increase in proportion of recreational injuries overall, and specifically bicycle, ATV/motorbike, and non-motorized wheeled vehicle injuries, is consistent with documented sale trends. It is possible that children had increased exposure to recreational equipment during the pandemic as they were not in school during the day. Adult data has indicated up to 20% of the US population who were previously not engaged in recreational activity changed this habit during the pandemic (Faulkner et al. 2021); these behavior changes could have also occurred in children. Closure of parks and limited travel during this time likely contributed to the decrease in playground and watercraft injuries. Additionally, likely due to the closure of schools and daycares, location of injury was more frequently in the home during the pandemic compared to prior.

There were significantly more male patients with recreational injuries overall, aligning with previously documented injury trends (www.cdc.gov/safechild/child_injury_data.html). However, there was no difference proportions of each sex injured in recreation during the pandemic compared to the years prior. Interestingly, more female patients were injured on bikes during the pandemic compared to before in our population, possibly due to more female patients riding bikes outdoors during COVID-19. One previous study similarly showed higher rates of cycling injuries requiring surgery in female patients during the pandemic; one proposed reason was anecdotal reports of decreased verbal and sexual harassment of female patients when biking due to decreased street traffic, leading to an increase in outdoor cycling (Faulkner et al. 2021). Across all studied recreational activities, more White patients were injured during the pandemic; this correlates with more White participants indicating use of recreational equipment (Taff et al. 2021). Higher rates of recreational injury were also seen in patients with commercial insurance (Shi et al. 2021), and those with a lower mean deprivation index, indicating a higher socioeconomic status. Notably, previous work in the early pandemic documented higher rates of hesitancy to seek pediatric emergency care in caregivers who were Black and of lower socioeconomic status, which may have impacted these results (Macy et al. 2021). This should be considered in an intersectional lens between race and socioeconomic status; in our population, this constellation of findings likely represents families that had increased access to and financial means to purchase recreational equipment used the devices more often during the pandemic, and thus, had higher rates of injury. Previous research has indicated children from households with higher socioeconomic status are more likely to engage in recreational activities, likely due to the time investment and cost, fitting with these results (Kemperman and Timmermans 2011).

There was no change in admission rates before and during the pandemic which may indicate no difference in injury acuity. However, with regard to bicycle injury, there were fewer presentations for abrasions and contusions compared to fractures and lacerations. This may be due to reduction in patients presenting to the PED, possibly to avoid COVID exposures for more minor injuries.

This research highlights a potential area for injury prevention. Direct counseling in the emergency department or primary care setting may be an ineffective means of injury prevention (Bar-on et al. 1998; Gittelman et al. 2003) but previous campaigns have demonstrated success with education, legislation, and safety equipment subsidies (Rivara et al. 1998; Thompson and Rivara 1998; Theurer and Bhavsar 2013). These data present an argument to increase funds for these types of interventions on a larger scale. For example, considerations to prevent recreational injuries may include promoting manufacturer required safety inserts, restraint and safety equipment laws, or free or low-cost helmet programs. An intersectional approach could be considered in any program implementation, considering our results with regard to race and socioeconomic status. For example, lower socioeconomic status is significantly associated with pediatric un-helmeted injuries, which is predictive of injury severity and associated with poor outcomes (Vittetoe et al. 2022); as such, when implementing injury prevention initiatives, it is important to identify which communities have the greatest need.

The retrospective data set is limited by missing or incorrectly listed information; we excluded data if unknown or missing values, but this may have altered results. The actual incidence of injuries due to recreational injuries is likely higher than documented here as patients in both time periods may not have had mechanism of injury documented. There is, however, no reason to think this under-documentation would have been different in prior to and during the pandemic. A third limitation that is worth noting is that patients choosing to present to the ED may have differed during COVID; patients may have gone to local urgent cares or primary care offices, or may have chosen to not pursue medical evaluation in the PED due to concern regarding risk of exposure to COVID in the PED. Additionally, this data was collected at a single-center and may not be generalizable to a broader population. A final limitation is that this study only looked at first few months of pandemic. Results may have been different if a longer period of the pandemic was studied.

## Conclusions

The COVID-19 pandemic impacted pediatric recreational injuries. Overall, our data suggests the pandemic resulted in a change in both the demographics of and equipment type implicated in recreational injuries. Further research is needed to determine future prevention efforts to curtail recreational injuries during a pandemic.

## Methods

This retrospective cohort study was conducted at a large free standing quaternary care children’s hospital PED with a level 1 trauma center. Data obtained electronically from the PED electronic medical records (EPIC Hyperspace Version 2021) included patients 5–14 years of age cared for between March 23 and Sept 1 of the years 2015–2020. These dates were chosen because the initial Ohio statewide stay-at-home orders for the COVID pandemic began March 23, 2020; we aimed to compare the first 6 months of the pandemic to the average of the same time period in the 5 years prior. This age group was selected based on standard injury reporting age groups (www.cdc.gov/safechild/child_injury_data.html). Additionally, children of this age are more likely to be unsupervised during play (Mack et al. 2012) and would have been affected by school closures.

ED encounters were included which met at least one PED International Classification of Disease-10 (ICD-10) code for injury including the use of common outdoor recreational equipment. We defined outdoor recreational activities as those pertaining to playgrounds, bicycles, ATVs/motorbikes, skateboards/scooters/roller skates/blades, trampolines, and watercraft (Additional file 1, ICD 10 codes V00–V06, V10–V19, V91, W09, Y93.44, Y93.55, V86). Pandemic year, 2020, was compared with the average of pre-pandemic years, 2015–2019. Patients with incomplete data for mechanism of injury were excluded. In addition, patients were excluded if their injuries were intentional or related to assault, were a complication of medical or surgical care, or were for any visit for the same injury within 7 days. In included encounters, ICD-10 codes classifying injuries by body region, describing nature of injury, and any external cause were collected for further analysis.

Data collected included patient demographics (age, sex, self-reported race, self-reported ethnicity, insurance type, and home zip code), type of recreational equipment used, location where the injury occurred (home, street/highway, daycare, school, public place, recreational or sport area, other/unknown), type of injury sustained, body part injured, and disposition (admit, discharge, other). Self-reported race options included White, Black or African American, Hispanic, or other (inclusive of Asian or Asian American, Native Hawaiian or other Pacific Islander, American Indian or Alaska Native, Multiple, Other, Unknown). Self-reported ethnicity options included Non-Hispanic, Hispanic, Unknown. Insurance type options included commercial, public, self-pay, and other/unknown. Data was extracted from EPIC by a senior research analyst based on age of patient and date of encounter and was then filtered by the investigators to include only pertinent mechanisms of injury. A deprivation index, a measure of socioeconomic status, calculated based on geocoded zip code data was assigned to each patient. Six weighted census variables are used to calculate the deprivation index with the resultant range of 0–1; higher scores indicate greater deprivation (Brokamp 2018; Brokamp et al. 2016, 2018).

Descriptive statistics were used to characterize the population. Comparisons of pandemic year, 2020, and the averages of the pre-pandemic years, 2015–2019, were made using Chi-squared analysis and student’s t-tests for categorical and continuous variables, respectively. A sub-analysis specific to bicycle injuries was also included. Statistical significance was defined as *p* < 0.05 for all tests. All statistical analyses were performed using IBM^®^ SPSS^®^ Statistics (Version 26).

### Supplementary Information

**Additional file 1**. Included ICD-10 Codes

## Data Availability

All data is maintained at Cincinnati Children’s Hospital Medical Center on password protected computers. The datasets generated and/or analyzed during the current study are not publicly available due containing identifying/contact information, but are available from the corresponding author on reasonable request.
