# Supplementary material for: Pediatric outdoor recreational injuries: another hidden concern during the COVID-19 pandemic
Source: Inj Epidemiol. 2023 Jun 29;10(Suppl 1):29. doi: 10.1186/s40621-023-00445-6 (PMC10311709; doi:10.1186/s40621-023-00445-6)
Supplement: Supplementary file 1 — Additional file 1. Included ICD-10 Codes [file 40621_2023_445_MOESM1_ESM.docx]

**Supplement 1: Included ICD-10 Codes**

| **Topic** | **ICD10 Code** |
| --- | --- |
| Trampoline | Y93.44 |
| Playground | W09 |
| Bike | Y93.55 |
| Skates | V00.1 |
| Pedal Cycle | V00 |
| Skateboard | V01 |
| Skateboard | V02 |
| Skateboard | V03 |
| Skateboard | V04 |
| Skateboard | V05 |
| Pedal Cycle | V10 |
| Pedal Cycle | V11 |
| Pedal Cycle | V12 |
| Pedal Cycle | V13 |
| Pedal Cycle | V14 |
| Pedal Cycle | V15 |
| Pedal Cycle | V16 |
| Pedal Cycle | V17 |
| Pedal Cycle | V18 |
| Pedal Cycle | V19 |
| Watercraft | V91 |
| Scooter | V00.14 |
| Motorized Scooter | V00.8 |
| Scooter | V06 |
| ATV/Motorbike | V86 |
